# Changes in population‐level alcohol sales after non‐medical cannabis legalisation in Canada

**DOI:** 10.1111/dar.14010

**Published:** 2025-02-03

**Authors:** Michael J. Armstrong, Rachael MacDonald‐Spracklin, Jennifer Xiao, Robert Talarico, Daniel T. Myran

**Affiliations:** ^1^ Goodman School of Business Brock University St. Catharines Canada; ^2^ Bruyère Health Research Institute Ottawa Canada; ^3^ Clinical Epidemiology Program Ottawa Hospital Research Institute Ottawa Canada; ^4^ ICES uOttawa Ottawa Hospital Research Institute Ottawa Canada; ^5^ Department of Family Medicine University of Ottawa Ottawa Canada; ^6^ School of Epidemiology and Public Health, Faculty of Medicine University of Ottawa Ottawa Canada

**Keywords:** cannabis legalisation, public health policy, alcohol, substitution, interrupted times series analysis

## Abstract

**Introduction:**

There is considerable interest in whether individuals substitute cannabis for alcohol and in legalisation's potential to reduce or increase alcohol‐attributable harms. This study aimed to determine whether non‐medical cannabis legalisation in Canada was associated with initial changes in population‐level alcohol consumption.

**Methods:**

This observational population‐based study described changes in alcohol sales in Canada between 2004 and 2022. We calculated annual changes in the per capita volume of pure ethanol sold in Canada. We used an interrupted time series approach to examine immediate and gradual changes in per capita price‐adjusted alcohol retailer sales value (CAD$) and beer producer sales volume (litres of product) after legalisation.

**Results:**

During 2004–2022, Canadians aged 15+ spent on average CAD $751 per year on alcoholic beverages containing 8.18 L of ethanol. Annual ethanol sales volumes decreased by 0.06 (95% confidence interval [CI] −0.08 to −0.04; *p* = 0.001) litres per capita annually for beer but increased by 0.05 (95% CI 0.04 to 0.07; *p* = 0.001) litres per capita annually for other beverages, leaving no significant trend for ethanol sales overall. Following non‐medical legalisation in October 2018, there were no immediate (−0.1%, 95% CI −1.3 to 1.1; *p* = 0.82) or gradual changes (−0.1% monthly, 95% CI −0.3 to 0.0; *p* = 0.12) in alcohol retailer sales.

**Discussion and Conclusion:**

Canada's non‐medical cannabis legalisation was not associated with significant changes in population‐level alcohol sales. These findings do not support the idea that cannabis legalisation may result in declining alcohol use and harms through the substitution of cannabis for alcohol.

## 1 INTRODUCTION

In the past decade, there has been a growing international movement towards liberalising cannabis policies and legalising non‐medical use. Canada became the second country to legalise non‐medical cannabis in October 2018, allowing adults to purchase and consume cannabis in various forms [1]. Additional countries are now proceeding with liberalisation, especially in Europe, where Germany legalised non‐medical cannabis use in April 2024 [2, 3]. Supporters have argued that liberalising cannabis may lead to reduced use and harms of other substances, particularly alcohol and opioids, via substitution (e.g. individuals might consume cannabis instead of alcohol) [4, 5, 6]. In contrast, critics have suggested that liberalised cannabis policies may lead to increased use or harm from such substances through complementary or co‐use [7, 8, 9]. These contradictory claims exist partly because high‐quality data are lacking regarding population‐level changes in alcohol use following cannabis liberalisation.

The limited existing literature on cannabis legalisation's relationship with population‐level alcohol sales presents conflicting findings. For example, one study found that alcohol sales decreased significantly within US states that introduced medical cannabis laws, compared to those that did not [10]. Conversely, a different study found no significant change in alcohol sales within US states that introduced non‐medical or medical cannabis laws [11]. Similar contradictions appear in the larger body of survey‐based research examining self‐reported alcohol purchasing or use. For example, in a study of three US states that allowed non‐medical use, self‐reported alcohol purchasing declined in two but increased in one, relative to states that did not authorise non‐medical use [12]. Meanwhile, a repeated cross‐sectional study found that self‐reported alcohol‐cannabis co‐use increased in US states that allowed non‐medical or medical cannabis use [8].

To date, two studies have examined population‐level alcohol sales changes associated with cannabis legalisation in Canada. The first study found a small negative correlation between medical cannabis sales and alcohol sales from April 2015 to September 2018, when non‐medical use was still illegal [13]. The second study analysed beer and spirit sales from October 2018 to February 2020, the first 17 months of non‐medical cannabis legalisation: it reported that canned beer sales decreased but spirit sales did not [14]; importantly, that study did not examine total alcohol sales or consider alternative reasons, including market factors, for the decrease in beer sales other than cannabis substitution.

An additional complication when examining alcohol's relationship with cannabis is that 18 months after legalisation, Canada began its emergency response to the COVID‐19 pandemic [15]. COVID‐19 pandemic disruptions in Canada led to increased mental distress [16, 17, 18], drinking to cope with emotional distress [19, 20] and greater sales of alcohol overall [21] but had no apparent impact on recreational cannabis sales trends [22]. With the additional influence of the COVID‐19 pandemic, disentangling the specific effects of cannabis legalisation on alcohol sales and consumption is challenging.

There is consequently a need for research involving high‐quality population‐level data to determine the potential substitution or complementarity relationship between alcohol and cannabis use. Canada's recent nationwide non‐medical cannabis legalisation presents a unique opportunity for such research. The current study examined changes in alcoholic beverage sales over time as a marker of population alcohol consumption following non‐medical cannabis legalisation in Canada. Specifically, we examined long‐term trends in annual alcoholic beverage sales from 2004 to 2022 and tested for changes in monthly sales after non‐medical legalisation but before COVID‐19 to isolate out early market effects from COVID‐19.

## 2 METHODS

### 2.1 Overview

This observational study investigated whether alcoholic beverage sales changed in Canada after non‐medical cannabis legalisation. We first used descriptive statistics to examine long‐term trends in annual sales broken down by beverage category. We then analysed short‐term changes in monthly sales after October 2018 using interrupted time series (ITS) models. The study did not require ethics review because it used publicly available aggregated data. The reporting followed Strengthening the Reporting of Observational Studies in Epidemiology guidelines.

### 2.2 Data sources

We obtained the annual sales of alcoholic beverages by retailers across Canada during each April‐to‐March fiscal year from 2004–2005 to 2022–2023 from Statistics Canada [23]. Alcohol sales were available by four beverage types: beers; wines; spirits; and “ciders, coolers, and other refreshment beverages” (hereinafter, “ready‐to‐drink alcoholic beverages [RTD]”).These sales were presented in terms of the absolute volume of litres of pure ethanol that were sold.

Seasonally adjusted monthly alcohol sales in Canadian dollars (CAD$) by provincial liquor boards and by beer, wine and liquor retailers (hereinafter, “alcohol retailer sales”) up to December 2022 were collected from Statistics Canada [24]. Alcohol retailer sales data were not available by beverage type. The value of alcohol sales from Statistics Canada is estimated through a combination of a monthly sample of private retailers (*n* = 1865 retailers on average per month in 2018) with monthly sales reports from provincial liquor authorities who are responsible for the wholesale of alcohol in most provinces in Canada. Retailers who are contacted by Statistics Canada to report their alcohol sales are legally required to report their sales to Statistics Canada. The data capture both off‐premise alcohol sales and on‐premise sales as drinking places in Canada obtain the majority of their alcohol through wholesale provincial liquor authorities or private retailers [21]. While the data have not been externally validated, they are considered of excellent quality by Statistics Canada.

Data on the total population of Canada as of 1 July of each year were from Statistics Canada, broken down by age, as well as the monthly consumer price indexes for alcoholic beverages sold through stores [25, 26].

Lastly, we obtained monthly beer sales in hectolitres (hereinafter, “beer producer sales”) between January 2012 and December 2022 from Beer Canada. Beer Canada is the industry association that represents Canada's large and mid‐sized beer producers, but not its small craft breweries. Its data cover approximately 90% of all beer consumed in the country [27].

### 2.3 Outcome measures

For annual sales, we took Statistics Canada's absolute ethanol volume for beer sold in each fiscal year and divided it by the population aged 15+ that year to calculate the litres of ethanol sold per capita in beer beverages. We similarly calculated the ethanol sold per capita for the other three beverage types. We added the values for spirits, wines and RTDs together to get a “non‐beer” subtotal and then added that to the beer values to get the total ethanol sold per capita each year. This yielded a time series with 19 annual measurements each.

For monthly sales, we took Statistics Canada's monthly dollar sales for alcohol retailers and cannabis retailers from January 2012 to December 2022 and then divided it by the population aged 15+ to get sales per capita. We then used Statistics Canada's consumer price indexes for alcohol and non‐medical cannabis to adjust those sales for price changes relative to December 2018 levels. This yielded price‐adjusted and seasonally adjusted monthly sales in CAD$ per capita for 132 months of alcohol retailer sales and 51 months of cannabis retailer sales.

We likewise divided Beer Canada's monthly producer volume sales from January 2012 to December 2022 by the population to get litres of beer sold per capita. We then processed that time series with the US Census Bureau's X‐13 ARIMA‐SEATS Seasonal Adjustment Program software to remove seasonal variations. This yielded seasonally adjusted monthly beer producer sales in litres of beer per capita for 132 months.

### 2.4 Statistical analysis

For annual sales, we calculated descriptive statistics to provide a long‐term overview of how alcohol sales have evolved. These included the total change in per capita sales between the 2004 and 2022 endpoints and the average annual change across all 19 observations. We calculated the latter using linear regressions of sales versus time.

For monthly sales, we performed segmented linear regression using ITS models to compare alcohol sales immediately before and after cannabis legalisation. We used the natural logarithms of per capita alcohol retailer sales and beer producer sales as our outcome measures so that we could report both sets of results as monthly percentage changes. Each regression equation included a constant plus three explanatory variables: the pre‐legalisation monthly sales trend; a level‐change variable measuring the immediate jump or drop in sales in October 2018; and a slope‐change variable measuring the change in monthly trend from October 2018 onward.

Before running the regressions, we checked whether linear modelling was appropriate by visually inspecting the time series plots [28] and calculating Wald tests for structural breaks. In the post‐legalisation period, there were increases in alcohol and beer sales from March 2020 onward during the COVID‐19 pandemic [21]. In the pre‐legalisation period, Wald tests indicated the presence of structural breaks (i.e. trend changes) in 2015 for alcohol retailer sales, plus in both 2015 and 2017 for beer producer sales; these appeared visually as curves in the time series. To avoid these non‐linear periods, we focused our regressions on the last 12 months before legalisation and the first 12 months afterward, i.e. from October 2017 to September 2019. Having the same number of observations in the pre‐ and post‐treatment periods potentially improved statistical power, whereas adding more observations risked confounding from the structural breaks pre‐legalisation or the pandemic disturbances post‐legalisation [28]. As a sensitivity analysis, we also ran models for the 17 months pre‐ and 17 months post‐legalisation.

We ran the regressions in Stata 18.5 software using the Prais‐Winsten method with robust standard errors to account for autocorrelation. This method has been recommended for ITS regressions [29]. After each regression, we used the delta method to compare a regression estimate of September 2019 sales, i.e. including the pre‐legalisation trend, level change and slope change, versus a counterfactual estimate of sales that would have been observed had legalisation not occurred [30], i.e. using only the pre‐legalisation trend. Our analysis interpreted statistical significance using 95% confidence intervals (CI) and *p* < 0.05.

## 3 RESULTS

### 3.1 Annual sales

**FIGURE 1:**
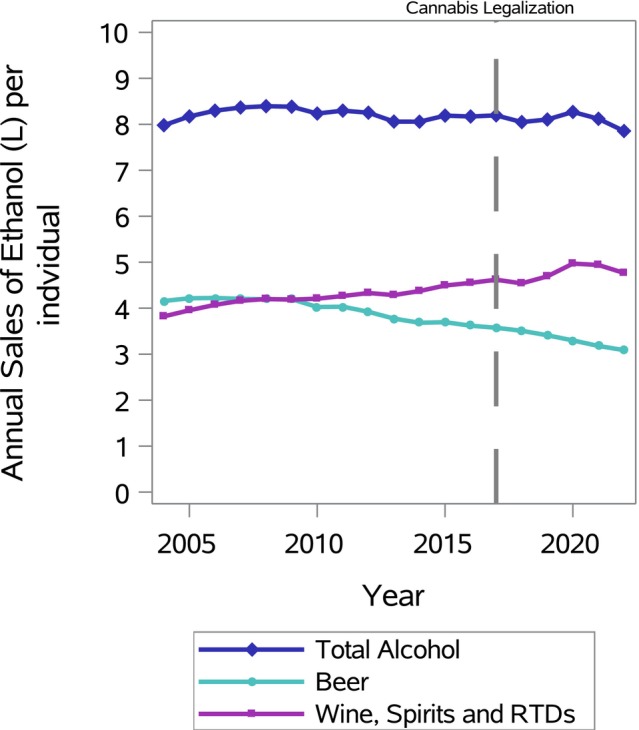
Annual alcohol sales in litres of ethanol per capita in total, for beer and non‐beer products, between 2004–2005 and 2022–2023. The vertical dashed line marks when cannabis became legal. Abbreviation: RTD, ready‐to‐drink alcoholic beverage.

During the 19‐year study period, Canadians aged 15+ consumed on average 8.18 L of pure ethanol per year and spent CAD $750.79 in 2022 dollars (see Table 1). During that 19‐year period, per capita ethanol sold in beer showed a significant decreasing trend of −0.06 (95% CI −0.08 to −0.04; *p* = 0.001) litres per capita annually; in total, it fell by 26%, from 4.16 to 3.09 L. Conversely, non‐beer ethanol sales showed a significant increasing trend of 0.05 (95% CI 0.04 to 0.07; *p* = 0.001) litres per capita annually; over 19 years, it rose by 25%, from 3.82 to 4.77 L. Consequently, total annual per capita ethanol sales showed no significant net trend. Beer's share of total ethanol sales therefore dropped from 52% to 39%. Figure 1 illustrates the diverging trends. Annual spending on alcohol also showed no significant trend over the period.

**TABLE 1:** Annual per capita sales between 2004/2005 and 2022/2023, in litres of ethanol for each alcohol product category, and in CAD$ for alcohol and non‐medical cannabis.

Product	Yearly average^a^	2004–2005	2018–2019	2022–2023	Total change^a^	Average annual change (95% CI)^a^
Ethanol sold (in litres)						
Alcohol	8.18	7.98	8.05	7.85	−0.13	−0.01 (−0.03, 0.01)
Beer	3.79	4.16	3.51	3.09	−1.07	−0.06 (−0.08, −0.04)
Non‐beer	4.39	3.82	4.54	4.77	0.94	0.05 (0.04, 0.07)
Wine	1.95	1.61	2.04	1.90	0.29	0.02 (−0.01, 0.04)
Spirits	2.11	2.01	2.11	2.19	0.18	0.01 (0.00, 0.02)
RTDs	0.32	0.20	0.39	0.67	0.48	0.03 (0.01, 0.04)
Dollars sold (in CAD$)						
Alcohol	750.79	687.48	760.23	742.52	55.04	3.17 (−1.10, 7.44)

Abbreviations: CI, confidence interval; RTD, ready‐to‐drink alcoholic beverage.

^a^ Yearly averages, total changes, and average annual changes cover the 19 years from 2004/2025 to 2022/2023 for alcohol, and the 5 years from 2018/2019 to 2022/2023 for cannabis.

### 3.2 Monthly sales

**FIGURE 2:**
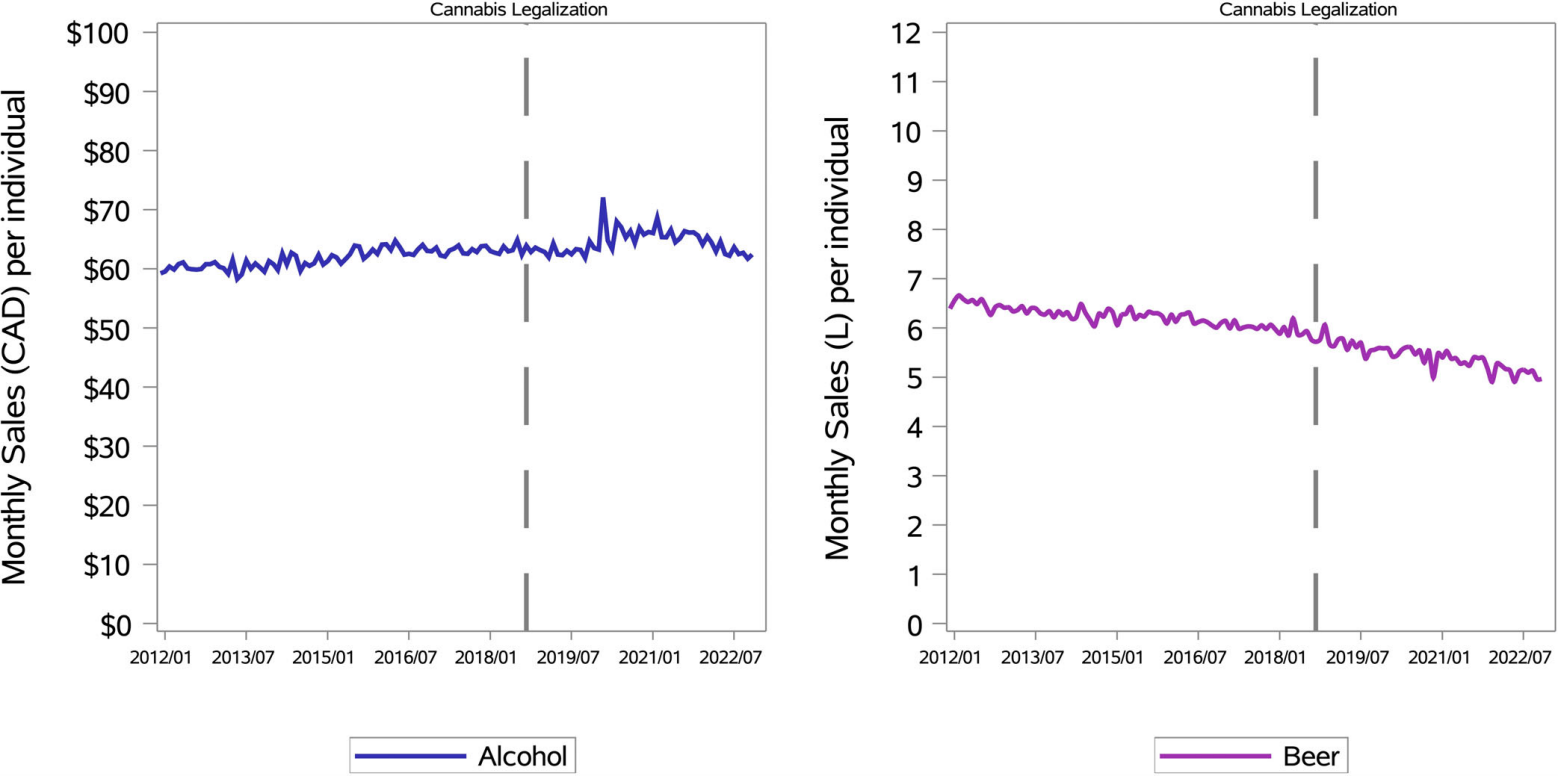
Monthly per capita alcohol retailer sales and cannabis retailer sales in dollars (left), and beer producer sales in litres (right), from January 2012 to December 2022. The vertical dashed line marks when cannabis became legal.

Figure 2 displays the monthly alcohol and beer sales from 2012 to 2022, while Table 2 summarises the monthly sales regressions.

**TABLE 2:** Regression results for monthly per capita alcohol and beer sales from October 2017 to September 2019.^a^

Regression parameter	Alcohol, percent (95% CI)	Beer, percent (95% CI)
Pre‐legalisation slope, per month	0.0 (−0.1, 0.2)	−0.3 (−0.4, −0.1)
Legalisation level change, immediate	−0.1 (−1.3, 1.1)	0.0 (−3.0, 3.1)
Legalisation slope change, per month	−0.1 (−0.3, 0.0)	−0.1 (−0.5, 0.2)
Difference from September 2019 counterfactual	−1.5 (−3.9 to 0.9)	−1.8 (−4.9 to 1.5)

Abbreviation: CI, confidence interval.

^a^ Interrupted time series regressions were run on the natural logarithm of alcohol retailer sales in CAD$ and beer producer sales in litres, per resident age 15+, with the output expressed as monthly percentage changes. *N* = 24 monthly observations: 12 months pre‐ and 12 months post‐legalisation.

**FIGURE 3:**
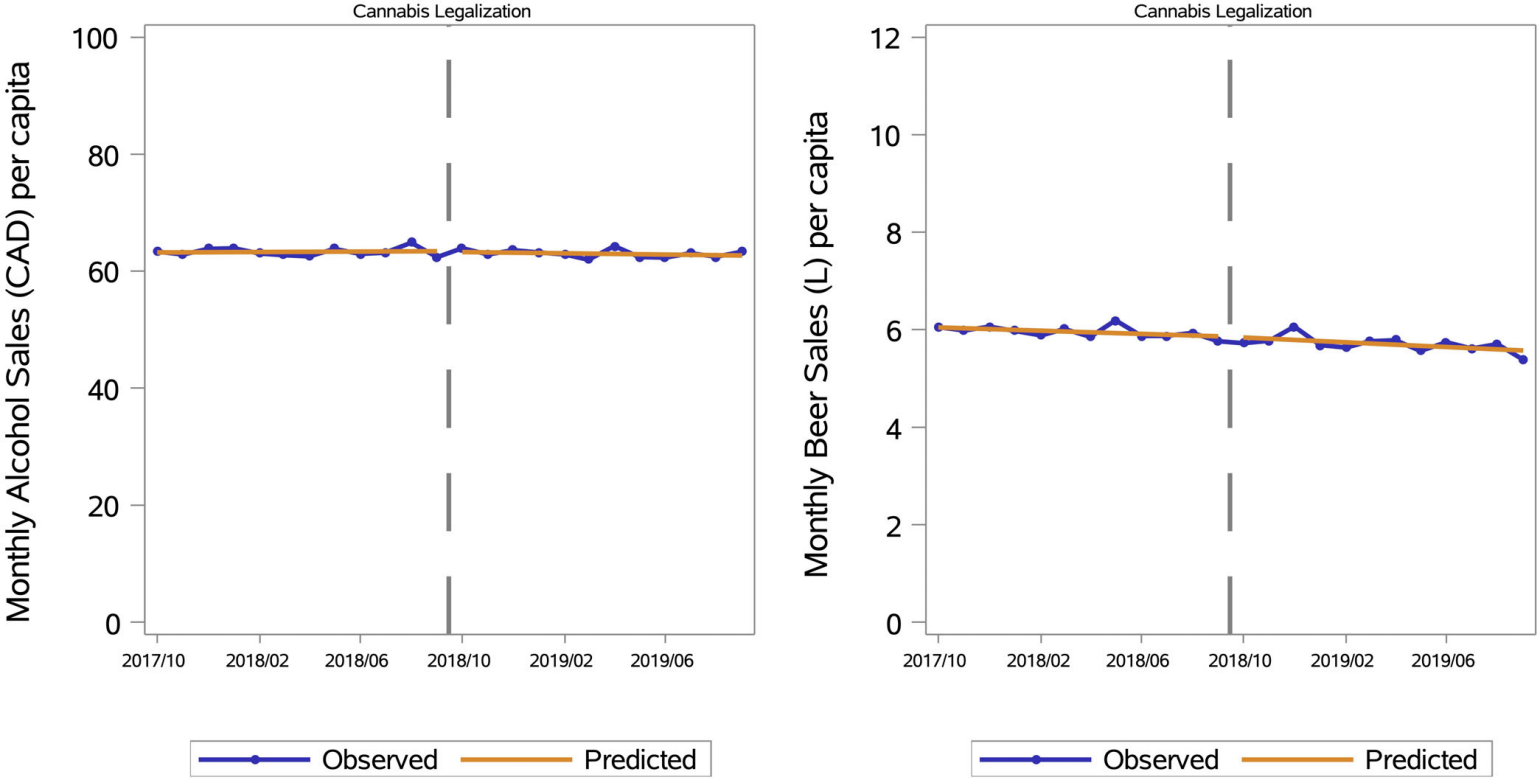
Seasonally adjusted monthly alcohol retailer sales in dollars per capita (left) and beer producer sales in litres per capita (right), plus the regression‐fitted values. The vertical dashed line marks when cannabis became legal.

For alcohol retailers, there was no significant sales trend before cannabis legalisation in October 2018 (0.0% average relative monthly change, 95% CI −0.1 to 0.2; *p* = 0.65). The immediate relative change in sales associated with legalisation (−0.1%, 95% CI −1.3 to 1.1; *p* = 0.82) and the relative monthly change thereafter (−0.1% monthly, 95% CI −0.3 to 0.0; *p* = 0.12) were also non‐significant. Consequently, the estimated difference in September 2019 between the regression‐fitted sales with legalisation and the counterfactual sales without legalisation was also non‐significant (−1.5%, 95% CI −3.9 to 0.9; *p* = 0.22) (see Table S1 for monthly per capita alcohol sales regression results over a 34‐month observation period). Figure 3 (left) illustrates these results by comparing the segmented regression lines to the observed sales values; both line segments are nearly horizontal (see Figure S1 for the same results with a narrower *Y* axis).

For beer producers, monthly sales before cannabis legalisation were decreasing by 0.3% per month (95% CI −0.4 to −0.1; *p* = 0.001). The immediate sales change associated with legalisation (0.0%, 95% CI −3.0 to 3.1; *p* = 0.99) and the trend change thereafter (−0.1% monthly, 95% CI −0.5 to 0.2; *p* = 0.42) were both non‐significant. Consequently, the estimated difference in September 2019 between the regression estimate and the counterfactual was also non‐significant (−1.8%, 95% CI −4.9 to 1.5; *p* = 0.29). Figure 3 (right) illustrates how the pre‐ and post‐legalisation line segments have very similar downward slopes.

## 4 DISCUSSION

This study was the first to examine population‐level changes in total per capita alcohol sales in Canada after non‐medical cannabis legalisation. We observed no significant changes in monthly alcohol sales or beer sales during the first 12 months post‐legalisation. We also saw no obvious changes in annual alcohol sales up to 4 years post‐legalisation; however, this longer‐term descriptive finding should be interpreted with caution given that it overlapped with COVID‐19's societal disruptions from February 2020 onward.

There has been long‐standing interest in whether individuals might substitute cannabis for alcohol [31], and cannabis legalisation has been proposed as a means to reduce societal alcohol use and associated harms [4, 5, 6]. A recent review suggested that there was more support for the substitution of alcohol for cannabis than for a complementary relationship or for no relationship; however, the review's findings were mixed [32]. In the USA, studies of state‐level medical and/or non‐medical cannabis laws have variously found subsequent decreases in alcohol sales [10], consumption increases in some states but decreases in others [12], increased alcohol‐cannabis co‐use [8] or no significant change [11]. In Canada, one prior study reported significant declines in canned beer sales following non‐medical cannabis legalisation and attributed that decline to consumers switching from beer to cannabis [14]. Our work adds to the existing literature by examining total alcohol sales in Canada and finding no association between non‐medical legalisation in Canada and changes in population‐level alcohol sales.

In contrast to that prior work in Canada, we found no significant change in beer sales associated with legalisation, as the post‐legalisation declines in beer sales were comparable to pre‐legalisation trends (i.e. consumers appeared to be switching away from beer at similar rates as before). In addition, our data indicate that when observing all alcohol types, legalisation is not associated with any significant increases or declines in alcohol sales. An examination of alcohol market trends suggests that a more plausible explanation for declining beer sales over time is changes within the alcohol market and consumer preference for different types of alcohol products. Specifically, RTDs were the fastest‐growing product type for seven consecutive years up to 2019 [33] and gained market share every year from 2010 to 2022 [34]. Therefore, the diverging trends in beer and non‐beer sales are more likely explained by consumers switching from beer products to the rapidly popularising RTDs rather than to cannabis. This highlights the importance of considering broader market trends when assessing cannabis legalisation's potential relationship with alcohol consumption.

Although our study suggests no meaningful population‐level changes in alcohol use immediately after legalisation, this relatively short period may not be representative of a more mature cannabis market. Complicating analysis of changes in alcohol use after legalisation, 18 months after non‐medical legalisation, Canada entered the COVID‐19 pandemic. The pandemic was associated with large disruptions in work, socialising and the daily lives of Canadians [35, 36, 37, 38], and studies have found increases in alcohol sales and self‐reported consumption, along with healthcare visits due to alcohol [21, 39, 40, 41]. Consequently, while our study found no meaningful population‐level changes in alcohol use up until 4 years after legalisation, the overlap of COVID‐19 and legalisation makes it challenging to understand longer‐term associations between legalisation and alcohol consumption. However, it is worth noting that by the end of our study period, Canadian's annual legal cannabis spending (CAD $192.99) had increased to the point where it was equivalent to one‐quarter of alcohol spending (CAD $742.52), without any observable change in alcohol sales [24]. Further studies of potential cannabis‐alcohol relationships are therefore needed, but will require rigorous designs along with consideration of contextual factors including the COVID‐19 pandemic [21] and alcohol regulatory changes [42].

### 4.1 Limitations

Our analysis has several limitations. First, we examined alcohol consumption in Canada at the population level, rather than at the individual consumer level. Consequently, while the findings suggest no changes in aggregate alcohol use after legalisation, it is possible that some sub‐populations or individuals might have reduced their alcohol consumption via substitution or increased it via co‐use. Second, the measure of alcohol consumption that is most strongly associated with population‐level use and harms, the per capita volume of ethanol sold, was only available annually. Consequently, we completed our monthly ITS analysis using per capita alcohol spending. Spending is not a perfect measure for usage, because changes in dollar values of alcohol sold might indicate shifts towards more or less expensive products, rather than changes in quantities consumed. Nonetheless, dollar values provide useful and timely insights into alcohol use and have been used in previous studies [21, 43]. Third, our ITS analysis of monthly alcohol sales found no significant changes during Canada's first 12 months with legal non‐medical cannabis. However, the cannabis market was still developing during that period. Sales were constrained by production shortages during the first 6 months [44] and thereafter grew as retail networks expanded [45, 46]. Consequently, it is possible that we detected no cannabis‐alcohol association because it was still taking shape. Finally, Canada's annual alcohol sales likewise displayed no apparent sign of meaningful change up to 2022–2023. However, as previously highlighted, this period overlapped the country's extensive COVID‐19 pandemic disruptions [15]. Consequently, it is possible that alcohol sales experienced cannabis‐related changes that were masked by concurrent pandemic‐related changes. Further research is therefore required to look for potential longer‐term cannabis‐alcohol relationships.

## 5 CONCLUSION

This population‐based study observed no changes in total alcohol sales or in beer sales associated with non‐medical cannabis legalisation in Canada. While a prior study reported that beer sales declined in Canada after non‐medical legalisation, our findings suggest those declines likely represented consumers switching from beer to RTDs, rather than to cannabis. While longer‐term monitoring is indicated, the present findings do not support non‐medical cannabis legalisation as a policy intervention to reduce alcohol use and its associated harms.

## AUTHOR CONTRIBUTIONS

All authors certify that their contribution to this work meets the standards of the International Committee of Medical Journal Editors.

## FUNDING INFORMATION

Dr. Myran was supported by a Canada Research Chair at the University of Ottawa Department of Family Medicine. The funders had no role in the design and conduct of the study; collection, management, analysis and interpretation of the data; preparation, review or approval of the manuscript; and the decision to submit the manuscript for publication.

## CONFLICT OF INTEREST STATEMENT

The authors have no interests to declare.

## Supporting information


**Data S1.**Supporting information.

## Data Availability

Data sharing is not applicable to this article as no new data were created or analyzed in this study.
