# Supplementary material for: Changes in population‐level alcohol sales after non‐medical cannabis legalisation in Canada
Source: Drug Alcohol Rev. 2025 Feb 3;44(3):811–9. doi: 10.1111/dar.14010 (PMC11886499; doi:10.1111/dar.14010)
Supplement: Supplementary file 1 — Data S1.Supporting information. [file DAR-44-811-s001.docx]

**Supporting Information**

This supplement has been provided by the authors to give readers additional information about the work.

Table of Contents

[Table S1 2](#_Toc179376998)

[Figure S1 3](#_Toc179376999)

Table S1. Regression results for monthly per capita alcohol sales from May 2017 to February 2020.^a^

| **Regression parameter** | **Alcohol,**  **percent (95% CI)** |
| --- | --- |
| Pre-legalisation slope, per month | 0.0% (-0.1, 0.1) |
| Legalisation level change, immediate | -0.6% (-1.8, 1.6) |
| Legalisation slope change, per month | -0.0% (-0.1, 0.1) |
| Difference from February 2020 counterfactual | -0.7% (-3.1 to 1.7) |

**^a^** Interrupted time series regressions were run on the natural logarithm of alcohol retailer sales in CAD$ per resident age 15+, with the output expressed as monthly percentage changes. N = 34 monthly observations: 17 months pre- and 17 months post-legalisation. CI, confidence interval.

# Figure S1. Seasonally adjusted monthly alcohol retailer sales in dollars per capita (left) and beer producer sales in litres per capita (right), plus the regression-fitted values.^a^

**
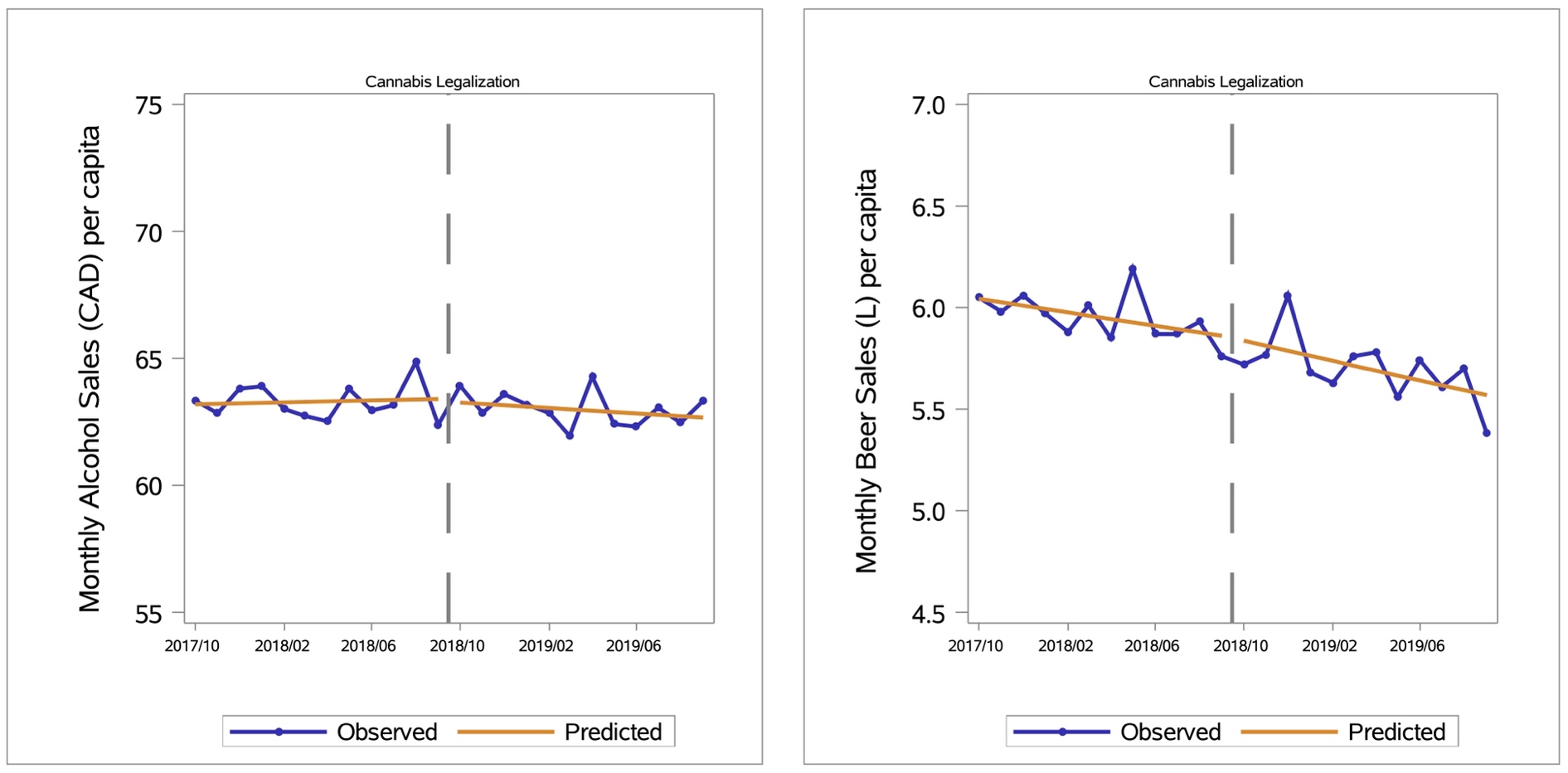
**

**^a^** The vertical dashed line marks when cannabis became legal.
